# Cytokine and human leukocyte antigen (HLA) profile for graft-versus-host disease (GVHD) after organ transplantation

**DOI:** 10.1186/s40001-016-0232-y

**Published:** 2016-10-12

**Authors:** Xinhua Chen, Xueqin Meng, Yuning Xu, Haiyang Xie, Shengyong Yin, Hongchun Li, Liming Wu, Shusen Zheng

**Affiliations:** 1 Key Laboratory of Combined Multi-organ Transplantation, Collaborative Innovation Center for Diagnosis and Treatment of Infectious Diseases, Ministry of Public Health, Hangzhou, China; 2 The Department of Hepatobiliary Surgery, The First Affiliated Hospital, Zhejiang University School of Medicine, 79 Qingchun Road, Hangzhou, 310003 Zhejiang China; 3 Key Laboratory of Hepatobiliary Disease in Shenzhen, Shenzhen, 518112 China

**Keywords:** Cytokine, Human leukocyte antigen (HLA), Graft-versus-host disease (GVHD), Transplantation, Multiplex immunoassay, High-throughput

## Abstract

**Background:**

Graft-versus-host disease (GVHD) after liver and kidney transplantation has high mortality and causes diagnostic challenges. This study aims to describe the cytokine and human leukocyte antigen (HLA) profile in the GVHD after liver and kidney transplantation.

**Methods:**

A high-throughput detection kit was applied and altogether 18 different cytokines were tested simultaneously. GVHD patients included 23 post-liver transplantation patients; 22 post-renal transplantation patients; The control patients include 22 hepatocellular carcinoma (HCC) patients without transplantation and 20 healthy controls. Their HLA characters were compared.

**Results:**

The full spectrum of cytokines was present. The inflammatory markers were activated significantly in liver transplantation. The level of inflammatory markers in liver transplantation was higher than that in renal transplantation, HCC or healthy controls. GVHD was associated with the HLA characters; HLA characters are involved in liver GVHD occurrence and act as risk factors.

**Conclusion:**

Our findings confirmed that the inflammatory cytokines play a pathogenic role in GVHD and can be used as early diagnostic markers. The HLA mismatch acts as a risk factor in liver transplantation to predict GVHD occurrence.

**Graphical abstract:**
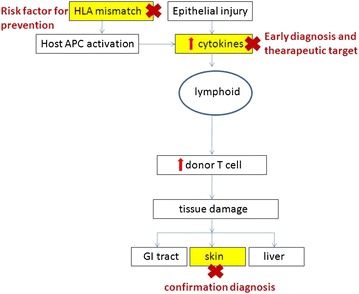
Diagnostic and therapeutic target for GVHD

## Background

Hepatocellular carcinoma (HCC) is the second leading cause of cancer-related death in the world [1, 2] and China accounts for almost half of HCC cases [2]. The liver transplantation is the curative treatment for HCC but post-transplantation graft-versus-host disease (GVHD) has caused mortality [3, 4]. The cytokine and HLA profile of GVHD can help understand the inflammatory background and the risk factors [5, 6]. The full profile of cytokine in the cytokine storms post GVHD is still lacking. In this study, we present a high-throughput cytokine exam for GVHD after liver and kidney transplantation. The association with HLA was also analyzed.

## Methods

### Patients

Altogether, 87 patients were included: 23 HCC patients with liver transplantation (LT); 22 renal transplantation (RT); 22 HCC without transplantation (HCC); and 20 healthy controls (control). Patients underwent organ transplant between January 2004 and December 2014.

### Measurement of cytokines by multiplex immunoassay

The multiple cytokines were measured by Multiplex Immunoassay Kit (Affymetrix, CA, USA). Briefly, 25 μL serum and standards were incubated with pre-mixed beads coated with antibodies. After washing, plates were incubated with the detection antibody and the reaction revealed with streptavidin–phycoerythrin. The beads were analyzed on a Luminex system (Bio-Rad, USA) for the concentration of the following 18 cytokines: IL-10, IL-17A, IL-21, IL-22, IL-23, IL-27, IL-9, GM-CSF, IFN-γ, IL-1b, IL-12, P70, IL-13, IL-18, IL-2, IL-4, IL-5, IL-6, TNF-α.

### Pathological confirmation of the skin biopsy

The pathological slides of the biopsy were reviewed and the pathological figures to support the diagnosis of GVHD was present. The skin sample was stained and read by two independent pathologists. The hematoxylin–eosin (H&E) was stained for the confirmation of GVHD.

### The anti-HLA antibody measurement

The HLA antibodies were measured by Luminex technology (One Lambda, Inc. Canoga Park, CA, USA). According to the manufacturer’s manual book, HLA-specific antibodies were identified using immune beads coated with purified HLA antigens. HLA antibodies in patient’s serum were bound to the antigens coated on the beads. The complex was then stained with R-phycoerythrin-conjugated goat anti-human IgG. The fluorescent emission of R-phycoerythrin was measured and then analyzed by HLA analysis software (Canoga Park, CA, USA.). All adjusted and normalized reactions that were above 500 were considered positive.

### Statistical analysis

The present data are expressed as mean ± SD. For statistical comparison of values, Student’s *t* test was used. *P* values less than 0.05 were deemed to indicate statistical significance. The SPSS one-way analysis of variance (ANOVA) was used to determine whether there were any statistically significant differences.

## Results

### The clinical presentation

The four groups are age-, sex-, and storage-time-matched. The typical symptoms were skin lesions. The liver function was not affected and the liver damage was not obvious. A nonspecific skin basal vacuolar changes, dyskeratosis in the epidermis were found. Diarrhea was the most common complaints due to the absorptive function loss caused by lymphocyte infiltration and destruction of the intestinal mucosa (Table 1).

**Table 1:** The clinical characteristics of patients

	Liver transplantation	Renal transplantation	HCC	Control
Number of patients	23	22	22	20
Recipient age (years)	54 ± 19	49 ± 12	58 ± 9	51 ± 21
Male/female	13/10	10/12	13/9	10/10
Time to onset (days)	253 ± 39	192 ± 51	/	/
BMI	22 ± 2.1	19 ± 3.4	20 ± 2.8	23 ± 3.2
AST (U/L)	56 ± 4	61 ± 2	45 ± 4	52 ± 3
ALT (U/L)	35 ± 2	28 ± 1	31 ± 3	31 ± 2
Total bilirubin (mg/dL)	0.95 ± 0.11	0.95 ± 0.16	0.95 ± 0.23	0.95 ± 0.37
Direct bilirubin (mg/dL)	0.45 ± 0.09	0.35 ± 0.14	0.47 ± 0.15	0.30 ± 0.17
Leukocytes (mil/mm^3^)	9.6 ± 2.1	4.8 ± 1.7	3.6 ± 0.4	4.5 ± 1.1
Haemoglobin (g/dL)	12 ± 2	11 ± 3	10 ± 2	14 ± 2

### Screening of cytokines

**Fig. 1:**
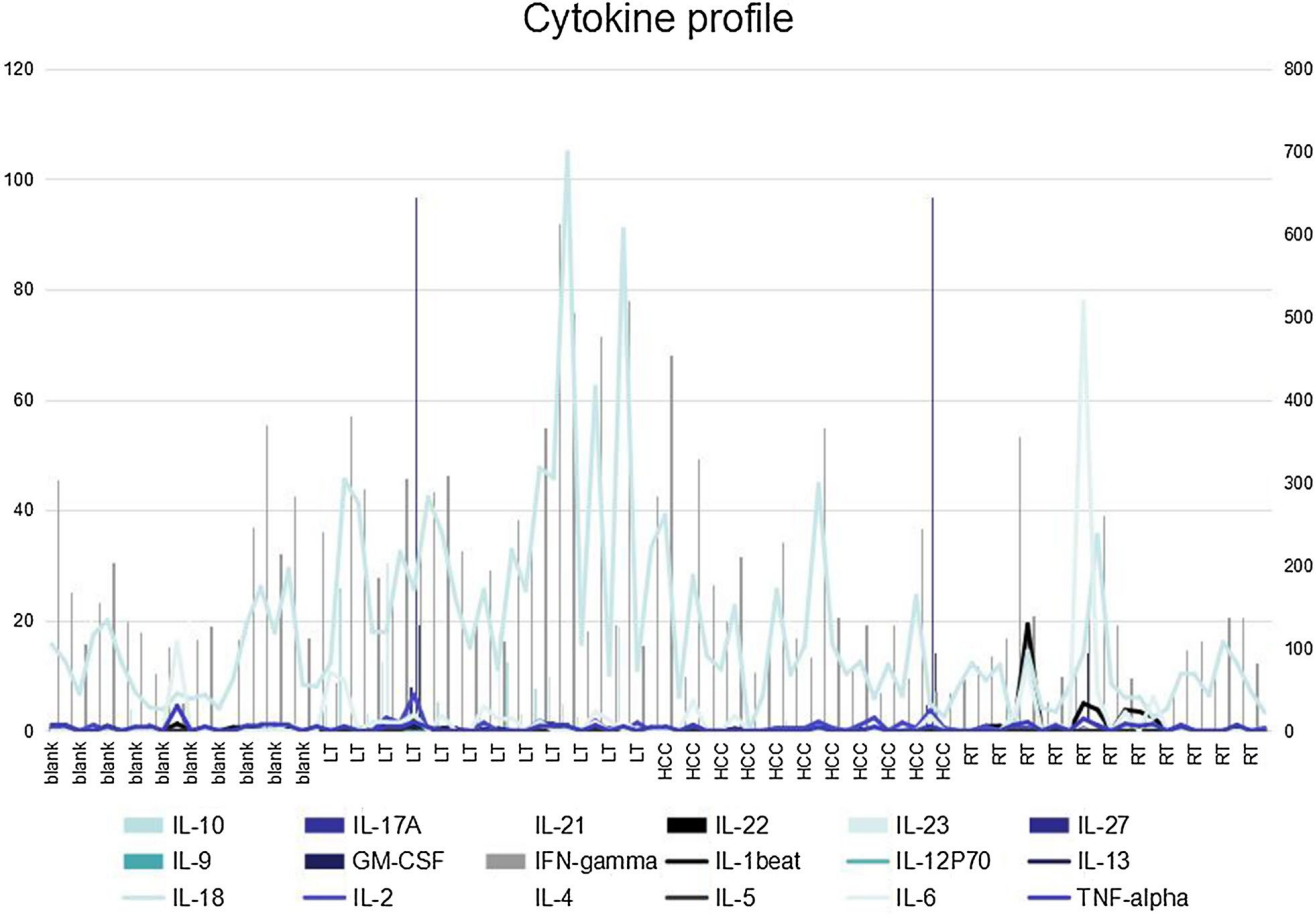
The cytokine profile measured by multiplex immunoassay. The multiple cytokines were measured by Multiplex Immunoassay Kit (Affymetrix, CA, USA) for the concentration of 18 cytokines (IL-10, IL-17A, IL-21, IL-22, IL-23, IL-27, IL-9, GM-CSF, IFN-γ, IL-1b, IL-12, P70, IL-13, IL-18, IL-2, IL-4, IL-5, IL-6, TNF-α). Among all the 18 screened cytokines, three cytokines IL-12, IL-18 and IFN-γ showed the significant increase and diagnostic value

Among all the 18 screened cytokines, three cytokines IL-12, IL-18 and IFN-γ showed a significant increase, and the diagnostic value varied from 0.5 (IL2) to 1.22 pg/mL (IL-18). The Luminex immunoassay measures cytokines in pg levels. Multiple cytokines can be tested in a single run by using a small volume of serum sample (Fig. 1).

### HLA identification

**Fig. 2:**
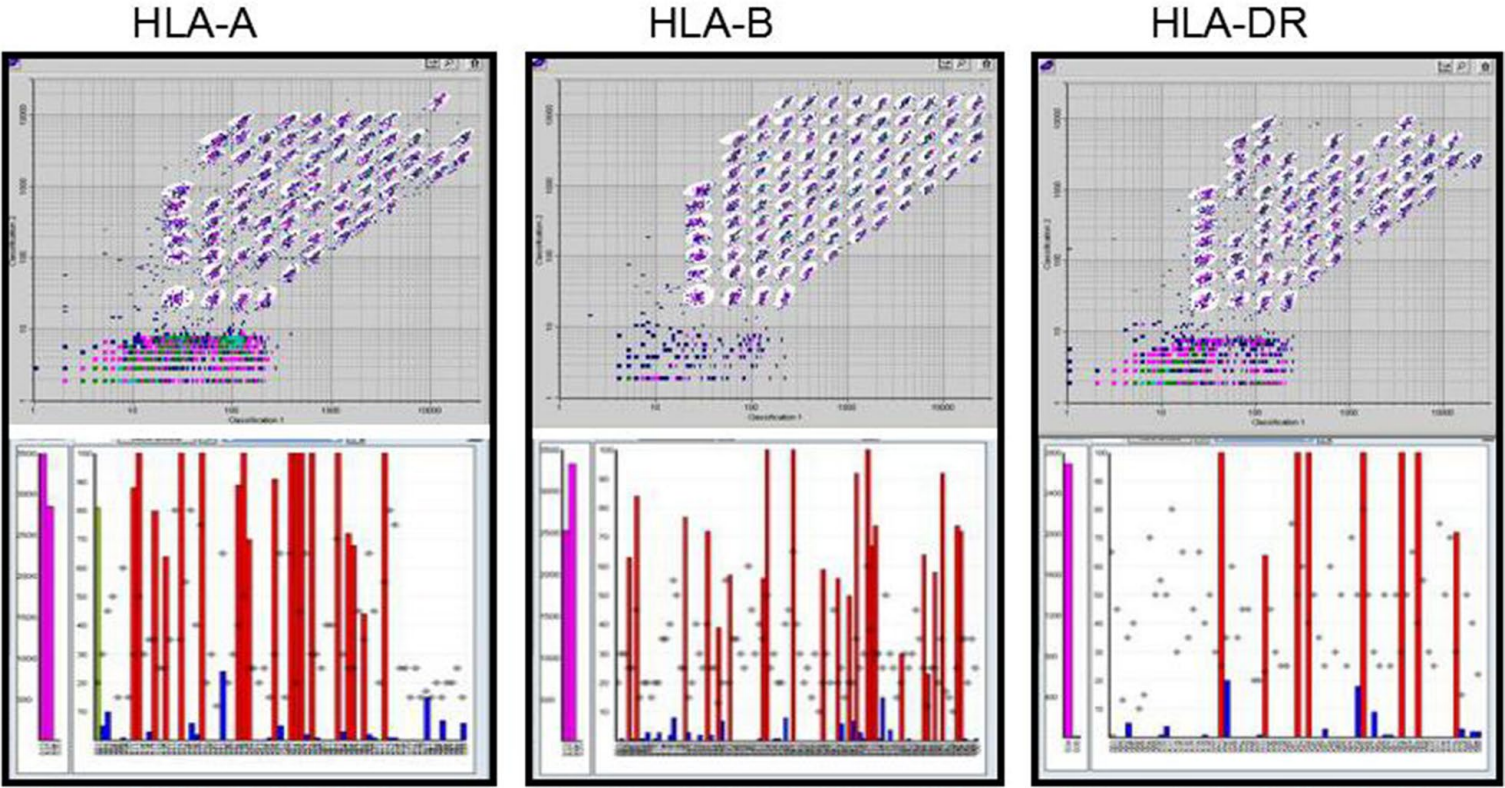
HLA profile in the recipient of GVHD post liver transplantation. The HLA antibodies was measured by Luminex system and the software from One Lambda, Inc. HLA specific antibodies were identified using immune beads coated with purified HLA antigens (*upper panel*). The fluorescent emission of antigen–antibody complex was measured and then analyzed (*lower panel*). All adjusted and normalized reactions that were above 500 were considered positive. HLA-A, B, DR were shown

HLA haplotype is shown in Fig. 2. There were donor–recipient HLA-mismatching in liver-transplanted patients. Renal transplantation (RT) requires a strict matching, so there was no HLA mismatching status in any of kidney case in our study. All of the 22 cases of renal transplantation (Fig. 2) had no mismatches at HLA-A, HLA-B, and HLA-DR loci. HLA-typing showed that the liver donor shared a single antigen with the patient, but the donor was heterozygous at other loci (A2, A24, B13, B46, DR12). Although liver transplantation (LT) dose not require a strict HLA compatibility as renal transplantation. Our result verified that the use of the HLA-mismatching donor can result in the risk of developing GVHD after LT.

### The pathology of skin lesion biopsy

**Fig. 3:**
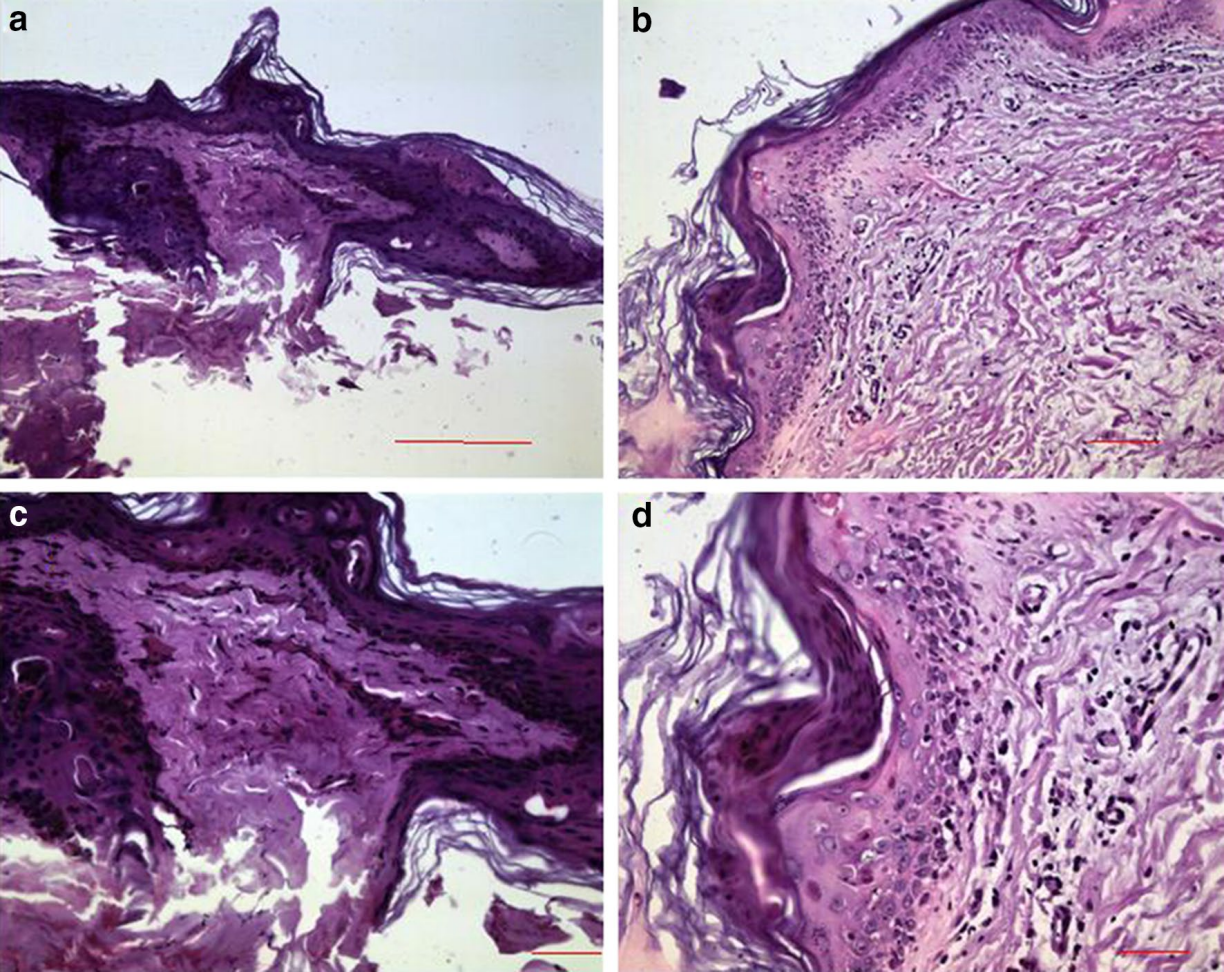
The pathology of skin lesion biopsy. The biopsy from skin on the right thigh (**a**). The pathology showed epidermal atrophy, excessive keratosis and parakeratosis in the epidermis (**b**) with significant dermal fibrosis and collagen (**c**). The lymphocytic infiltration was seen but not significant (**d**). The inflammatory cells infiltrate and invade into the epithelium (**b**). Diagnosis: skin squamous cell dyskeratosis associated with dermal chronic inflammatory cell infiltration

The biopsy from skin on right thigh of GVHD patient was shown (Fig. 3a). The pathology showed epidermal atrophy, excessive keratosis and parakeratosis in the epidermis (Fig. 3b) with significant dermal fibrosis and collagen (Fig. 3c). The lymphocytic infiltration was seen but not significant (Fig. 3d). The dermal perivascular inflammatory cell infiltration was found invading into the epithelium (Fig. 3b). Diagnosis: skin squamous cell dyskeratosis associated with dermal chronic inflammatory cell infiltration.

Different from kidney transplantation, which requires a strict donor–recipient HLA matching to improve long-term prognosis, liver transplantation can tolerate the HLA-mismatching to some degree. The incompatibility between donor and recipient might inhibit the host immune function. Whether HLA-B matching and the other HLA loci may impact long-term survival in liver transplantation is still debating [12, 13]. We use it as a risk factor rather than an absolute evaluation biomarker.

## Discussion

There are three basic requirements formulated by Billingham in 1966 for the development of GVHD: the graft must contain immunologically competent cells; the recipient must express tissue antigens that are not present in the transplant donor; and the recipient must be incapable of mounting an effective response to eliminate the transplanted cells [7–9]. Our results proved that GVHD post liver transplantation involved HLA mismatch and inflammatory cytokine release.

Examining the levels and interplay of cytokines can help to identify GVHD biomarkers and medical intervention targets [10]. However, the complicated interactions of cytokine network in GVHD can only be understood when the full cytokine profile is present [11]. Multiplex immune-assays can detect multiple cytokines with a small amount of patient’s serum (only 25 μL) with good parallel among different cytokines. The multiplex luminescence immunoassay can simultaneously detect antibody activity to 18 antigens in a single 96-well plate. The exam can be performed automatically in a centralized laboratory. The results indicate that after GVHD onset, an exaggerated response of cytokines IL2, IL18, IFN-γ may predispose to GVHD. They can be used to predict the occurrence of GVHD in transplant recipients. High levels of the three cytokines above correlated with the development of GVHD. The results suggest that measurement of IL2, IL18 and IFN-γ is of predictive value. Beside IL2, IL18 and IFN-r, the baseline of other 15 cytokines were also present (IL-10, IL-17A, IL-21, IL-22, IL-23, IL-27, IL-9, GM-CSF, IL-1b, IL-12P70, IL-13, IL-4, IL-5, IL-6, TNF-α). These results together provide a full cytokine profile and further substantiate the complex cytokine cascade that is initiated by GVHD.

Another notable finding in our study is the cytokine difference between GVHD post liver transplantation and renal transplantation. The GVHD and cytokine situation in liver transplantation and renal transplantation is quite different. IL2, IL18 and IFN were all lower in RT group than other groups. To address this difference, we have to add an additional HLA-typing experiment to explain the reason. The renal transplantation requires a very strict HLA matching. So, all renal transplantation cases were HLA-matched. The GVHD in renal transportation and inflammation response were not as high as that in liver transplantation. The strict HLA match in renal transplantation might be the possible reason. We added a typical case of LT to show its HLA-typing. HLA-mismatching allows patient to survive in liver transplantation without acute rejection. But it did increase the risk of GVHD in long term.

The immune mechanism on GVHD involves T cells and B cells. However, it is becoming increasingly focused on host-derived molecules that result from tissue incompatibility, e.g., HLA-mismatching [12]. Our result showed that HLA contribute to the occurrence of GVHD and then the rejection reaction was so wide that it caused skin damage. An improved understanding of these pathways may reveal novel therapeutic targets to decrease GVHD and increase long-term survival.

One major risk factor for the development of GVHD after LT was determined: human leukocyte antigen (HLA) matching between the donor and the recipient; Donor–recipient compatibility for HLA-B was identified as a significant risk factor for the development of GVHD. An accurate diagnosis of GVHD depends on histological and immuno-histochemical proof [13]. In our study, the pathological in skin include lymphocyte infiltration, basal cell vacuolization and the keratinocyte resembling. The differential diagnosis excluded the bacterial, viral and fungal causes of gastrointestinal symptoms. Then, the patients were given standard treatment of increased immune suppression and antibody preparations. Immune suppressants are a necessary right after transplant surgeries, preventing the rejection-caused new organ function failure. But the long term use of immune suppressants can cause many side effects such as infection, cancer, diabetes, GVHD compared to the general population. The proper withdrawal or minimize the use of immune suppression can help the immune system reconstitute itself.

## Summary

Our study provides a comprehensive platform for GVHD diagnosis. The HLA-mismatching works as a risk factor for the physician to suspect the high possibility of occurrence. Cytokine profile can be used for early diagnosis. Skin biopsy can be used as confirmation.

## Conclusion

Our findings confirmed that the inflammatory cytokines play a pathogenic role in GVHD and can be used as early diagnostic markers and future therapeutic targets. The HLA mismatch acts as risk factor in liver transplantation to predict GVHD occurrence.

## Abbreviations

HLA: cytokine and human leukocyte antigen
GVHD: graft-versus-host disease
HCC: hepatocellular carcinoma
