# Erratum to: Cytokine and human leukocyte antigen (HLA) profile for graft-versus-host disease (GVHD) after organ transplantation

**DOI:** 10.1186/s40001-017-0275-8

**Published:** 2017-10-03

**Authors:** Xinhua Chen, Xueqin Meng, Yuning Xu, Haiyang Xie, Shengyong Yin, Hongchun Li, Liming Wu, Shusen Zheng

**Affiliations:** 1 Zhejiang University, School of Medicine, The First Affiliated Hospital, Ministry of Public Health, Key Laboratory Combined Multiorgan Transplant, Collaborative Innovation Center for Diagnosis and Treatment of Infectious Diseases, 79 Qingchun Road, Hangzhou, 310003 Zhejiang People’s Republic of China; 2 Zhejiang University, School of Medicine, The First Affiliated Hospital, The Department of Hepatobiliary Surgery, 79 Qingchun Road, Hangzhou, 310003 Zhejiang People’s Republic of China; 3 Key Laboratory of Hepatobiliary Disease in Shenzhen, Shenzhen, 518112 People’s Republic of China

## Erratum to: Eur J Med Res (2016) 21:38 DOI 10.1186/s40001-016-0232-y

Following publication of the original article [1], the authors requested a correction in the presentation of their institutions as they appear on the Web of Science. Specifically, it should be emphasized that the institutions belong to Zhejiang University. The correct presentation of affiliations is given in this erratum.
